# Decoding Imagined 3D Arm Movement Trajectories From EEG to Control Two Virtual Arms—A Pilot Study

**DOI:** 10.3389/fnbot.2019.00094

**Published:** 2019-11-14

**Authors:** Attila Korik, Ronen Sosnik, Nazmul Siddique, Damien Coyle

**Affiliations:** ^1^Intelligent Systems Research Centre, Ulster University, Derry, United Kingdom; ^2^Hybrid BCI Lab, Holon Institute of Technology, Holon, Israel

**Keywords:** brain-computer interface (BCI), imagined 3D arm movements, online motion trajectory prediction, multiple linear regression (mLR), filter-bank common spatial Patterns (FBCSP), electroencephalography, virtual robotic arm

## Abstract

**Background:** Realization of online control of an artificial or virtual arm using information decoded from EEG normally occurs by classifying different activation states or voluntary modulation of the sensorimotor activity linked to different overt actions of the subject. However, using a more natural control scheme, such as decoding the trajectory of imagined 3D arm movements to move a prosthetic, robotic, or virtual arm has been reported in a limited amount of studies, all using offline feed-forward control schemes.

**Objective:** In this study, we report the first attempt to realize online control of two virtual arms generating movements toward three targets/arm in 3D space. The 3D trajectory of imagined arm movements was decoded from power spectral density of mu, low beta, high beta, and low gamma EEG oscillations using multiple linear regression. The analysis was performed on a dataset recorded from three subjects in seven sessions wherein each session comprised three experimental blocks: an offline calibration block and two online feedback blocks. Target classification accuracy using predicted trajectories of the virtual arms was computed and compared with results of a filter-bank common spatial patterns (FBCSP) based multi-class classification method involving mutual information (MI) selection and linear discriminant analysis (LDA) modules.

**Main Results:** Target classification accuracy from predicted trajectory of imagined 3D arm movements in the offline runs for two subjects (mean 45%, std 5%) was significantly higher (*p* < 0.05) than chance level (33.3%). Nevertheless, the accuracy during real-time control of the virtual arms using the trajectory decoded directly from EEG was in the range of chance level (33.3%). However, the results of two subjects show that false-positive feedback may increase the accuracy in closed-loop. The FBCSP based multi-class classification method distinguished imagined movements of left and right arm with reasonable accuracy for two of the three subjects (mean 70%, std 5% compared to 50% chance level). However, classification of the imagined arm movement toward three targets was not successful with the FBCSP classifier as the achieved accuracy (mean 33%, std 5%) was similar to the chance level (33.3%). Sub-optimal components of the multi-session experimental paradigm were identified, and an improved paradigm proposed.

## Introduction

Brain-computer interface (BCI) research targets movement-free communication between a human user and an electronic device using information encoded in electrophysiological activity of the brain without involving neuromuscular pathways. Two significantly different approaches are commonly used to achieve continuous control on electronic devices using non-invasively recorded electrophysiological correlates of motor function (van Gerven et al., 2009), i.e., sensorimotor rhythms (SMR) based multi-class classification and movement/motion trajectory prediction (MTP) (in some applications movement direction classification). The first approach uses SMR based multi-class classification to assign control commands to different cognitive task-specific brain activity patterns, normally using discrete time windows of information during the movement or movement imagery (Pfurtscheller et al., 2006; Morash et al., 2008) to achieve multi-functional control over objects in real (LaFleur et al., 2013) or virtual space (Royer et al., 2010). For example, in Yu et al. (2016) two distinct motor imagery tasks (imaginary movements of the left and right hands) was used to generate a multi-task car control strategy comprising of engine start, moving forward, turning left, turning right, moving backward, and stopping the engine. Furthermore, Feng et al. (2018) calls attention to a subject-specific time latency between onset of the motor imagery task and a feature window that maximizes the accuracy of the classification method. Thus, using a subject-specifically optimized latency of the feature window can lead to an increased performance of the classification method. The second approach (MTP) decodes the trajectory of an executed, observed, or imagined limb movement, i.e., the time-varying limb coordinates or velocity vectors are estimated. While the application of SMR based BCIs using multi-class classification to a wide range of devices may be possible, MTP BCIs focus on prosthetic, robotic, or virtual limb control. The non-invasive MTP approach was introduced by Georgopoulos et al. (2005) for magnetoencephalography (MEG) in 2005, and it was applied to electroencephalography (EEG) by Bradberry et al. (2010) in 2010. Most MTP BCI experiments have decoded single upper limb movements toward different targets in 3D space (Bradberry et al., 2010; Choi, 2013; Yeom et al., 2013). Finger movement (Paek et al., 2014), drawing tasks (Georgopoulos et al., 2005), or complex movements, such as walking (Presacco et al., 2011) or drinking a glass of water (Heger et al., 2012) have also been investigated using noninvasively recorded brain activity (EEG or MEG).

Although MTP research aims at estimating 3D trajectories of imagined movements, most MTP studies to date have focused on prediction of executed movement trajectories. Prior to this study, only a few MTP studies have presented results for non-executed movements, e.g., movement observation in one (Ubeda et al., 2017) or two orthogonal 2D plane(s) (Kim et al., 2015), prediction of imagined movements in horizontal or vertical directions (Ofner and Müller-Putz, 2015), estimating the speed of an imagined grasp task (Yuan et al., 2010), or decoding 3D trajectory of imagined arm movements (Korik et al., 2018b).

Regarding movement observation, Ubeda et al. in a recent study (Ubeda et al., 2017) decoded 2D trajectories of executed and observed movements with multiple linear regression (mLR). However, in Ubeda et al. (2017) imagined movement (motor imagery) task was not involved. Although Kim et al. decoded 3D trajectory of executed and imagined arm movements with multiple linear regression (mLR) and kernel ridge regression (KRR) methods (Kim et al., 2015), the motor imagery task was performed in parallel with the observation of a human volunteer or robot performing 3D arm movement. In Ofner and Müller-Putz (2015) the motor imagery task was synchronized with a metronome, the required imagery movement was not presented during the motor imagery task, and the task involved performing imagery of arm movements in vertical and horizontal directions of a 2D plane and not a complex 3D movement. In contrast with (Kim et al., 2015; Ofner and Müller-Putz, 2015; Ubeda et al., 2017) wherein the executed, observed, or imagined movements were decoded from the low delta EEG oscillations, typically in the 0.5–2 Hz frequency range, Yuan et al. (2010) showed that, imagined clenching speed of the left and right hand can be decoded from power of the EEG signal in mu and beta bands using mLR. Finally, our recent study (Korik et al., 2018b) showed that, 3D trajectory of imagined arm movements could be decoded from EEG with an mLR model using bandpower of mu, low beta, high beta, and low gamma EEG oscillations, and that low delta band oscillations, although providing a limited amount of information for decoding movement trajectories, provided significantly lower decoding accuracy than the other aforementioned bands for both executed and imagined 3D arms movements.

Although the final goal of MTP research is a real-time control of a virtual or artificial limb in a closed-loop using the 3D trajectory of imagined movements, to date, MTP studies have focused on offline decoding methods. As reviewed in Wolpert et al. (1998), motor learning is a complex process wherein the cerebellum plays an important role in a closed-loop application. Therefore, real-time feedback enables the brain to adapt to the required cognitive state and it can be used to improve the performance during a multi-session learning process (Wolpert et al., 2011). Müller-Putz et al. (2018) presented two closely related studies to classify, in closed-loop, six natural single different joint movements of the same arm and three different grasp types from motor-related cortical potentials (MRCPs) in a narrow 0.3–3 Hz band. In other studies such different applications were studied as the control of a cursor in 2D (Wolpaw and McFarland, 2004), classification of finger movements in closed-loop (Lehtonen et al., 2008; Hotson et al., 2016), open and grasp of a prosthetic hand (Fukuma et al., 2015), controlling an upper-limb exoskeleton for stroke survivors (Bhagat et al., 2016), use of a lower limb exoskeleton during flexion and extension (Liu et al., 2017) and walking task (Lee et al., 2017), and using a robotic arm to reach target objects in a 2D plane (Baxter et al., 2013; Meng et al., 2016). However, none of these studies presented real-time control of a prosthetic, robotic, or virtual arm using the 3D trajectory estimation of imagined arm movements decoded from EEG.

Thus, following our offline studies (Korik et al., 2015, 2016a,b,c), a pilot study presented here was aimed at studying the real-time control of two virtual arms in a closed-loop using 3D trajectory of imagined arm movements decoded from EEG. The difference in the target classification accuracy calculated from predicted 3D trajectories in runs using different levels of assistance for calculating visual feedback indicated that an over-balanced negative visual feedback during online control may have had an impact on accuracy during online task performance (Alimardani et al., 2014). As target classification accuracy provided only limited success for separating three corresponding targets per arm, key elements of the experimental paradigm have been reviewed and recommendations for modifications are proposed to support future work targeting online control with EEG-based imagined 3D trajectory decoding.

## Methods

### Subjects

Three right-handed volunteers (males, mean age 37 years) participated in the experiment, which was conducted at the BCI lab at the Intelligent Systems Research Centre (ISRC), Ulster University, United Kingdom. All subjects were healthy without any reported medical or psychological illness and/or medication, and had normal or corrected to normal vision. Prior to the research beginning, subjects were presented with information about the experimental protocol and were asked to read and sign an informed consent form to participate in the study, which was approved by the Ulster University research ethics committee (UREC). Subject 1 was experienced in classical motor imagery BCI control while the other two subjects were naïve in motor imagery BCI experiments.

### Experimental Paradigm

**Figure 1:**
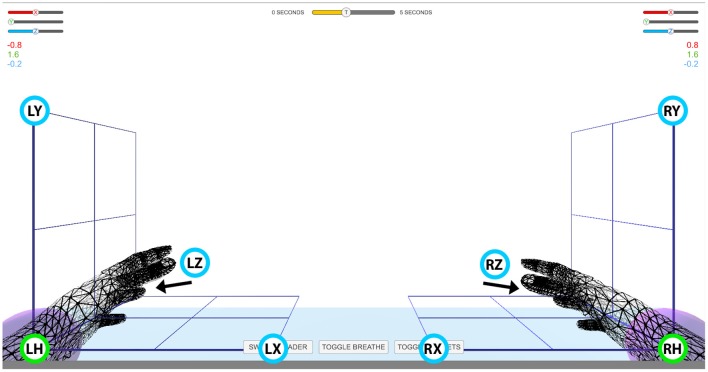
Virtual arm layout. Experimental setup for controlling virtual arms in 3D space. Green circles with labels LH and RH denote left and right home position, respectively. Blue circles with labels LX, LY, LZ and RX, RY, RZ denote target positions for the left (L) and right (H) hand in the horizontal (X), vertical (Y), and depth (Z) planes, respectively. The movement of the virtual arms was restricted to an area indicated with the cubic grid. The numbers above the grid on the left and right side of the screen indicate Cartesian coordinates of the virtual arm. The slider bars above the Cartesian coordinates and the time indicator bar at top of the screen were not used in this study.

The experiment comprised seven sessions, each session lasting ~2 h including EEG preparation time. In each session, the subjects were seated in an armchair positioned 1.5 m in front of a Fujitsu Siemens B22W-5 ECO 22″ LCD monitor. Targeted and decoded movements were displayed on the screen using two virtual arms controlled by the Unity 3D Game Engine. The positions of the three targets for each hand were selected in three orthogonal directions from the view angle of the corresponding home position, that is in horizontal (x), vertical (y), and depth (z) directions from the left or right home position. This experimental setup enabled standardized trajectories, i.e., the same orthogonal distance between the home position of the virtual hand and each of the corresponding targets (Figure 1). Before the beginning of the experiments, subjects were requested to look forward and maintain a constant head position, avoid teeth grinding and to minimize unnecessary movements during task performance. They were also asked to try to avoiding eye blinks during imagined movement cycles.

Each of the seven sessions comprised three parts: an offline part without feedback, an online part providing assisted feedback (Equation 6), and an online part with direct feedback. Details of the offline and online paradigms are described below.

#### Offline Paradigm

**Figure 2:**
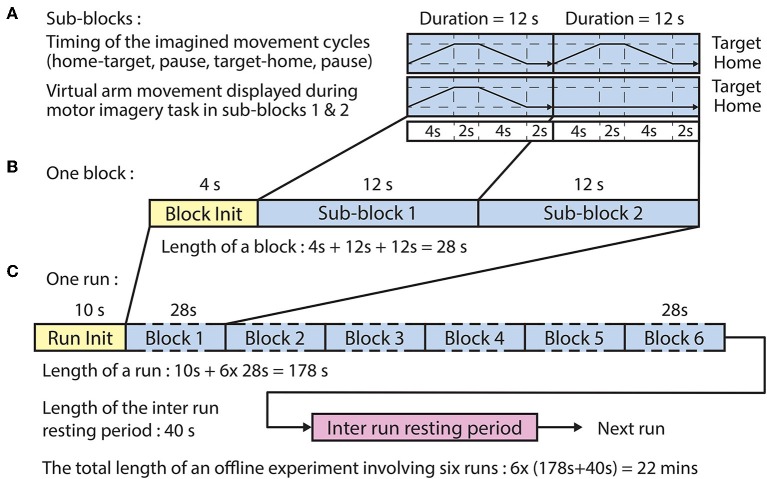
Timing of the offline paradigm. **(A)** Timing of the imagined movement cycles in sub-blocks 1 and 2 between a home position and one of the three corresponding target positions. In both sub-blocks, the subject performed the same motor imagery movement cycle that comprised home-to-target (4 s), pause at target (2 s), target-to-home (4 s), and pause at home (2 s) intervals. However, visual representation of the required movement was only displayed in sub-block 1. **(B)** A block comprised a block initialization period and two sub-blocks. **(C)** A run comprised a run initialization period and six blocks corresponding to the six targets in a randomized order (the six targets comprised three targets per arm).

The offline paradigm (Figure 2) comprised six runs, each run comprising six blocks wherein each block comprised two sub-blocks, each composed of four epochs:

- Home-to-target: a movement period between the home position and a target position synchronized with a 4 s auditory cue (6 kHz tone)
- Pause-at-target: a 2 s pause at the target position without an auditory cue
- Target-to-home: a movement period between the target position to the home position synchronized with a 4 s auditory cue (4 kHz tone)
- Pause-at-target: a 2 s pause at the home position without an auditory cue.

Ten seconds before commencing each run, a voice message was played to inform the subject about the upcoming run. Four seconds before commencing each block, a vocal message informed the subject about the actual task which was for the left hand: “move left hand to right,” or “move left hand to top,” or “move left hand forward.”

Subjects were asked to kinesthetically imagine their own arm moving in the trajectory illustrated by the virtual arm during the first imagined movement cycle (sub-block 1). In the second imagined movement cycle (sub-block 2), the virtual arm was idle at the home position and the subject was instructed to perform an imagined arm movement in the same trajectory as it was performed during the first imagined movement cycle (i.e., in sub-block 1). Subjects were asked to kinesthetically imagine their own arm moving in the trajectory illustrated by the virtual arm in the first trial (sub-block 1).

**Figure 3:**
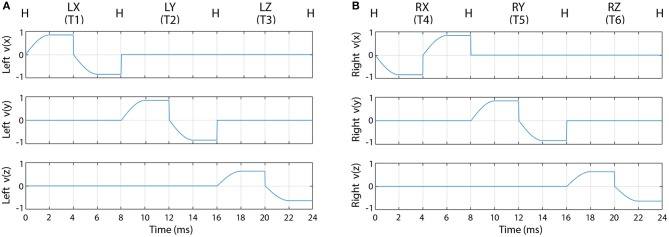
Velocity trajectories used for the offline part of the experiment. The figure represents the velocity of the left **(A)** and right **(B)** virtual hand movement during the first sub-block (pause periods are not presented). Label “H” indicates the left **(A)** or right **(B)** hand at the home position. Labels LX, LY, LZ and RX, RY, RZ indicate X, Y, and Z, positions of the left **(A)** and right **(B)** hand, respectively.

For the first sub-block, when the required movement was displayed on the screen, the speed of the virtual arm in the forward direction started from zero, reached a maximal value at half way between the home and the target position, and kept constant until the virtual arm reached the target position. After the pause epoch at the target position, the speed of the virtual arm in the backward direction started from zero, reached a maximal value at half way between the target and the home position, and kept constant until the virtual arm reached the home position. Velocity trajectories of the virtual arm during all possible movement periods are illustrated in Figure 3.

The BCI was trained using a dataset recorded during the offline runs of the session. The time duration of each sub-block was 12 s (Figure 2A), and the time duration of each block, consisting of a voice message and two sub-blocks, was 28 s (Figure 2B). The order of the imagined movements toward different targets (corresponding to LX, LY, LZ, RX, RY, and RZ directions, illustrated in Figure 1) was randomized in each run and distributed over the six blocks. The time duration of each run was 178 s (Figure 2C), and the inter-runs resting period lasted 40 s. Thus, the total duration of the offline part of the session comprising six offline runs was 22 min.

#### Online Paradigm

**Figure 4:**
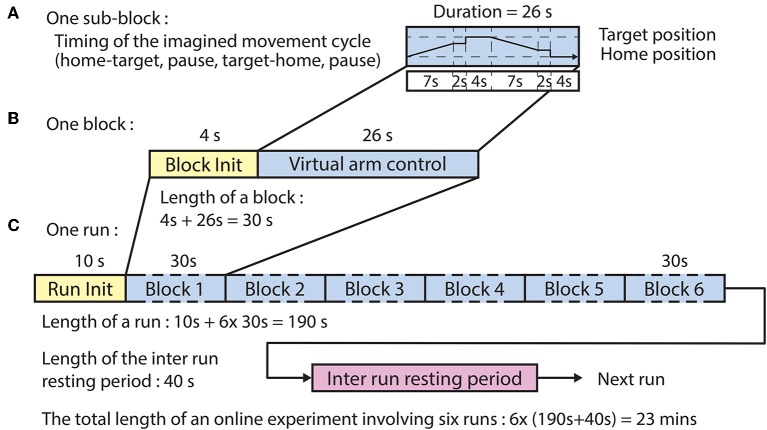
Timing of the online paradigm. **(A)** Timing of the virtual arm control between the home position and one of the three corresponding target positions. The imagined movement cycle comprised home-to-target movement (7 s), pause [at movement end-point (2 s) + at target (4 s)], target-to-home movement (7 s), and pause [at movement end-point (2 s) + at home (4 s)] intervals. **(B)** A block comprised a block initialization period and an imagined movement cycle. **(C)** A run comprised a run initialization period and six blocks corresponding to the six targets in random order (the six targets comprised three targets per arm).

The online experimental paradigm using assisted and direct feedback (Figure 4) followed the same structure comprising six runs wherein each run comprised six blocks.

Ten seconds prior to the commencing of each run a voice message was played to inform the subject about the upcoming run. Four seconds prior to the commencing of each block, a vocal message informed the subject about the actual task which was for the left hand: “move left hand to right,” or “move left hand to top,” or “move left hand forward.” For each block, the corresponding virtual arm was controlled from a home position toward a target position (forward movement) and back to the home position (backward movement). The forward movement was synchronized with a 7 s auditory cue (6 kHz tone), which was muted earlier if the virtual hand reached a position where the virtual hand was closer to the target positions than 20% of the distance between the home and actual target positions. At the end of the movement, the virtual arm was held at its current location for a short pause (2 s) after which the virtual hand relocated at the target position for a 4 s pause. Next, the backward movement was synchronized with a 7 s auditory cue (4 kHz tone), which was muted earlier if the virtual hand reached a position where the virtual hand was closer to the home positions than 20% of distance between the target and actual home positions. At the end of the movement, the virtual arm was held at the current position for a short delay (2 s) after which the virtual hand was re-located at the home position for a 4 s pause. Thus, the maximum duration of each movement block was 30 s (Figure 4B) consisting of the block initialization voice message (4 s) and the movement cycle with maximal duration of 26 s (Figure 4A). The order of the imagined movements toward different targets was randomized in each run and distributed over the six blocks. The maximum duration of each run was 190 s (Figure 4C), and subsequent runs were separated by an inter-run resting period lasting 40 s. Thus, the maximum duration of the online control comprising six online runs, was 23 min for both types of virtual arm control method which described below at “Online signal processing for MTP” headline.

### Data Acquisition

**Figure 5:**
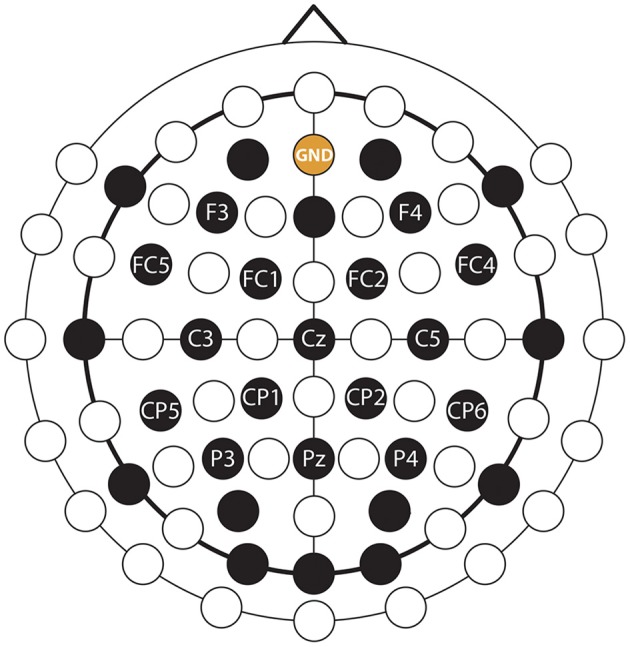
EEG montage. EEG and ground channels used for this study are labeled. Non-labeled black channels were also recorded, but were not used in this study (see the explanation in the text body).

EEG was recorded from 30 channels and electrooculography (EOG) was recorded from two channels using an EEG system with 32 active EEG sensors with two cross-linked 16 channels g.BSamp bipolar EEG amplifiers and two AC type g.GAMMbox (g.GSamp 16 channels, 2017). The EEG reference electrode was positioned on the left earlobe. The EEG was amplified (gain: 20,000) and sampled (A/D resolution: 24 Bits, sampling rate: 120 samples/s). The ground electrode was positioned over the AFz electrode location according to the international 10/20 EEG standard. The electrodes placement configuration was designed to cover multiple brain areas as the same EEG cap was used for multiple, parallel running studies wherein different cortical areas were studied. In this study, the participants were asked to perform imagined kinesthetic movements, thus, EEG channels near the sensorimotor area were more likely to provide task-related information during imagined kinesthetic movements (Neuper et al., 2005) than signals over other cortical areas. To that end, only 16 channels with a homogeneous distribution near the sensorimotor cortical areas were used in this study (Figure 5).

Data synchronization among EEG dataset (EEG signals) and kinematic dataset (velocities and positions of the virtual arms during the offline and online runs) was ensured by time stamps stored simultaneously with the data recording in both datasets. The communication between the EEG data acquisition software in Simulink, the code of the experimental protocol in Visual Basic, and the virtual arm application in Unity 3D Game Engine was handled by a user datagram protocol (UDP) based communication.

### Offline Signal Processing for MTP

The BCI was calibrated in each session using the EEG-kinematic dataset recorded at the beginning of each session during the offline run. The optimal BCI architecture was used for predicting imagined arm movements in the same session during the online runs.

#### Pre-processing

The quality of the recorded EEG was inspected manually for all channels, and EEG channels with high-level noise were removed from further processing. To reduce common mode artifacts, EEG was re-referenced using a CAR filter as described in Equation (1) according to McFarland et al. (1997) and Lu et al. (2013):

(1) $$\begin{array}{l} V_{i}^{CAR}=V_{i}^{ER}-\overset{N}{\underset{j=1}{\sum }}V_{j}^{ER}/N \end{array}$$

where $V_{i}^{ER}$ is the potential between electrode *i* and the reference and *N* is the number of EEG channels used for signal processing.

Our prior experiments showed that, bandpower of mu, low beta, high beta, and low gamma EEG encode more information from an imagined arm movement than slow cortical potentials (SCPs) in the low delta band (Korik et al., 2018b), therefore, in this study, band power of 8–12 Hz (mu), 12–18 Hz (low beta), 18–28 Hz (high beta), and 28–40 Hz (low gamma) oscillations were used for decoding imagined kinesthetic movements from EEG.

The time-varying band power was calculated based on the re-referenced EEG signals using the four non-overlapped EEG bands (described above) using a sliding window of 250 ms width with an 8.33 ms time lag between two adjacent windows. The band power within a time window was calculated by averaging the square values of the band-pass filtered EEG potentials as done in the previous offline study (Korik et al., 2018b) and described in Equation (2):

(2) $$\begin{array}{r} B_{fn}\left[t\right]=\frac{\sum_{m=1}^{M}{\left({P\left(m\right)}_{fn}\left[t\right]\right)}^{2}}{M} \end{array}$$

where *B*_*fn*_[*t*] is the band power value calculated from EEG channel *n*, using band-pass filter *f* , within a 250 ms width time window with offset *t*. *M* is the number of samples within a time window and *P*(*m*) is the *m*th band-pass filtered sample within the time window.

The trajectories of the virtual arm movements displayed during the first sub-block (Figure 3) were used as a template of expected imagined movements performed in both sub-blocks (i.e., when virtual arm movement was displayed and when it was not displayed on the screen). Thus, as no visual feedback of the hand was given in the second sub-block, it was guaranteed that the EEG signal was not affected by movement observation related artifact.

#### Kinematic Data Prediction

The kinematic data estimation module was prepared based on Bradberry et al. (2010) and applied in previous MTP studies (Korik et al., 2015, 2016a,b,c, 2018b). To enable decoding information from more than one EEG band, here we use features from multiple bands. The BCI was trained separately for decoding velocity of the left and right hands in three orthogonal directions. The core equation of the kinematic data estimation module is described in Equation (3):

(3) $$\begin{array}{r} v_{ij}\left[t\right]=a_{ij}+\overset{N}{\underset{n=1}{\sum }}\overset{B}{\underset{f=1}{\sum }}\overset{L}{\underset{k=0}{\sum }}b_{ijnfk}S_{jnf}\left[t-k\right]+\varepsilon_{ij}\left[t\right] \end{array}$$

where *a*_*ij*_ and *b*_*ijnfk*_ are regression parameters that learn the relationship between the input *S*_*jnf*_ [*t* − *k*] time-varying feature vector and the output *v*_*ij*_ [*t*] time-varying kinematic data. *v*_*ij*_ [*t*] contains the three orthogonal velocity components of the arm at left and right hand joint positions, *S*_*jnf*_ [*t* − *k*] is a standardized temporal difference of EEG band power values in frequency band *f* at sensor *n* at time lag *k*. Index *i* denotes spatial dimensions in the 3D orthogonal coordinate system, index *j* denotes joints at left and right hand positions, *N* is the number of sensors, *L* is the number of time lags, and ε_*ij*_ [*t*] is the residual error. The embedding dimension (or model order) is the number of time lags plus one (*L* + *1*), i.e., the number of time lagged samples that are selected from each channel for estimating kinematic data at time point *t*. The standardized difference for using band power features is described in Equation (4):

(4) $$\begin{array}{r} S_{jnf}\left[t\right]=\;\frac{B_{jnf}\left[t\right]}{\sigma_{B_{jnf}}} \end{array}$$

where *B*_*jnf*_ [*t*] is the value of the input time-series at time *t* (i.e., a band power value) and σ_*B*_*jnf*__ is the standard deviation of *B*_*jnf*_.

The time lag between two estimations (i.e., the time lag between two samples in the predicted kinematic dataset) was set to 25 ms to match the 40FPS refresh rate of the virtual arm. As a final post-processing step, jitters in the predicted kinematic data were reduced with a smoothing filter using a moving window with nine samples width (i.e., 200 ms).

#### Optimal Parameter Selection and Training for the Online MTP BCI

**Figure 6:**
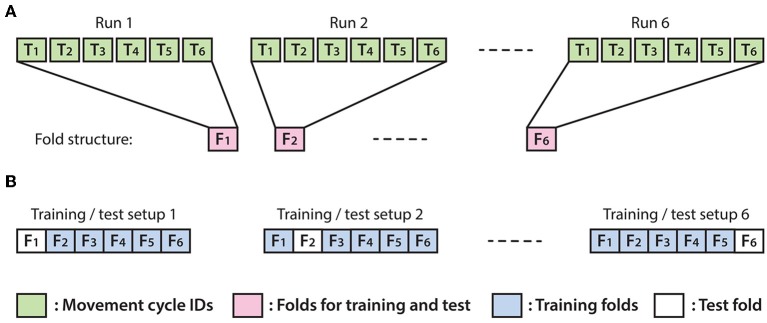
Structure of the applied leave-out-one cross-validation (CV) technique. **(A)** Each fold was associated with a run and comprised data from the second sub-block in which the virtual arm did not move (imagined arm movements were synchronized with an auditory cue). **(B)** Illustration of six possible options for separating training and test data using 6-fold leave-out-one CV.

The optimal EEG channel set, time lag and embedding dimensions (i.e., number of time lags +1) providing the highest test accuracy (Box 1) were selected with a recursive method described in this section. The test accuracy was measured using the leave-out-one cross-validation (CV) technique. The number of the folds used for CV was assigned to six and each fold was matched one of the six offline runs (Figure 6). Thus, the test dataset in the test folds was never used for training. The decoding accuracy of the test data was calculated as described in Box 1.

Box 1 **Accuracy metrics.**

As accurate control of the virtual arm requires minimizing the 3D distance between reconstructed (predicted) and expected (target) coordinates of the controlled joint (i.e., length of the 3D position error vector), the mean value of this error value was calculated over each movement time-point (see details below):

Target (and reconstructed) kinematic data in each fold involve six movement cycles, each movement cycle performed between a home position and one of the three corresponding target positions, and comprises a movement interval in the forward direction (home to a target) and a movement interval in the backward direction (target to home) resulting in twelve trials per fold. As the speed of the moving virtual arm was normalized to a constant value in the online application, the length of the target and reconstructed velocity vectors was also normalized to the value of one as described in Equation Box 1 (1), below:

Box 1 (1) $$\begin{array}{l} v_{i}^{*}=\frac{v_{i}}{\sum_{i\;=1}^{3}{\left(v_{i}\right)}^{2}},{{v^{\prime }}_{i}}^{*}=\frac{{v^{\prime }}_{i}}{\sum_{i\;=1}^{3}{\left({v^{\prime }}_{i}\right)}^{2}} \end{array}$$

where $v_{i}^{*}$ and ${v^{\prime }}_{i}^{*}$ are the normalized target and normalized reconstructed velocity vector components, respectively, and *v*_*i*_ and ${v^{\prime }}_{i}$ are the original (non-normalized) target and original reconstructed velocity vector components, respectively, according to the spatial dimension *i*.

The $v_{i}^{*}$ (normalized target) and ${v^{\prime }}_{i}^{*}$ (normalized reconstructed) velocity values were converted into relative coordinates by integrating velocity values from sample points in each trial, separately, according to the three spatial dimensions as described in Equation Box 1 (2), below:

Box 1 (2) $$\begin{array}{l} x_{tim}=\frac{\sum_{\omega \;=1}^{m}v_{ti\omega }^{*}}{m},{x^{\prime }}_{tim}=\frac{\sum_{\omega \;=1}^{m}{v^{\prime }}_{ti\omega }^{*}}{m} \end{array}$$

where *x*_*tim*_ targeted relative coordinates and ${x^{\prime }}_{tim}$ and reconstructed relative coordinates were computed from $v_{ti\omega }^{*}$ normalized target and $v_{ti\omega }^{\prime *}$ and normalized reconstructed velocity values, respectively, involved in the corresponding trial *t* according to spatial dimension *i*∈*[1,2,3]*. The sample points in a trial are indexed by *m*.

Each of the twelve movement trials within a fold was converted into relative coordinates, separately, using the first sample in each trial as a reference point of zero for the actual trial. The relative coordinate based error calculation eliminated a cumulative error in coordinates that could have resulted by integrating the error resulting from velocity values over multiple trials. The reconstruction error within a trial was computed by the mean value of 3D distance between reconstructed and expected (target) coordinates across the entire trial as describe in Equation Box 1 (3), below:

Box 1 (3) $$\begin{array}{l} \varepsilon_{t}=\frac{\sum_{m\;=1}^{M}\sqrt{\sum_{i\;=1}^{3}{\left(x_{tim}-x_{tim}^{\prime }\right)}^{2}}}{M} \end{array}$$

where *e*_*t*_ is the reconstruction error in trial *t* calculated from 3D distance of *x*_*tim*_ relative target and $x_{tim}^{\prime }$ is the relative reconstructed coordinates in trial *t*. *M* and *m* are the number of the samples and their index in a trial, respectively, and the spatial dimensions are indexed with *i*.

The mean value of ε_*t*_ reconstruction error calculated from twelve trials in six movement cycles within a test fold was used as a metric to measure decoding accuracy in the test fold.

In the first step of the recursive parameter selection method, input parameters of the kinematic data estimation module (according to Equation 3) comprised all of the sixteen preprocessed channels. In each recursive step, the channel that achieved the lowest score in the previous step was omitted. The estimation module was trained for each combination of the investigated time lag parameters (i.e., investigated time lags: 50, 100, 200 ms, and number of the time lags: 1, 2, 4, 8). The test accuracy (described in Box 1) was calculated and stored, separately for each time lag parameter option. The range of the investigated time lag parameters was assigned using experience gained from previous offline MTP studies involving decoding imagined arm movements from bandpower of EEG (Korik et al., 2016b, 2018b).

EEG channels used in the current recursive step have been ranked using a similar linear regression parameter scoring method described by Sanchez et al. (2004) and Bradberry et al. (2010). The channel scores were obtained as described in Equation (5):

(5) $$\begin{array}{r} R_{jnf}\;=\;\frac{\sum_{k\;=\;0}^{L}\sqrt{\sum_{i\;=1}^{3}b_{ijnfk}^{2}}}{L+1} \end{array}$$

where *R*_*jn*_ is the score of channel *n* according to velocity estimation of joint *j* (left or right hand) while *b*_*ijnfk*_ variables are regression parameters obtained from the training (indices *i, j, n, f, k* are defined in Equation 3). Scores obtained from six test folds in four frequency bands were averaged and the channels were ranked based on the final scores for each of the two hand joints, separately. The channel with the lowest rank was marked and omitted from the following recursive step. The recursive method involving training, testing and channel ranking steps was repeated until all channels were ranked.

The number of the EEG channels selected for the online application was between six and twelve. An EEG channel set that scored the highest decoding accuracy rates for each arm was selected for the online BCI runs along with the corresponding time lags and linear regression parameters.

A general overview of the signal processing steps used for the offline analysis is illustrated in the offline section of **Figure 8**.

### Online Signal Processing for MTP

The online part of the experiment followed a protocol illustrated in Figure 4. EEG data acquisition and preprocessing (using a Simulink module designed for online signal processing) was similar to the module, which applied to the offline analysis. The velocity prediction for left and right hand in three orthogonal spatial dimensions was realized with six separated linear regression modules. Each module was calibrated using an optimal BCI architecture (i.e., EEG channel set, time lag, and number of the time lags) and linear regression parameter setups resulted from the offline analysis. The sampling rate of the estimated kinematic data was down-sampled to 40 Hz, and a smoothing filter using a nine samples width (i.e., 200 ms) moving window was applied as used for the offline analysis. The communication between the Simulink module, Visual Basic software, and the Unity 3D Game Engine was handled with UDP using the same protocol that was applied to the offline part of the experiment. Predicted velocity vectors were normalized as described in Equation Box 1 (1) and hand positions were displayed on the screen using assistance or without assistance according to the online paradigm. In runs involving assistance, the displayed coordinates were calculated using an assistance-level specific linear combination of the targeted and predicted velocity vectors as described in Equation (6):

(6) $$\begin{array}{r} v_{assisted}^{*}\left[t\right]=\rho v_{target}^{*}\left[t\right]+\left(1-\rho \right)v_{predicted}^{*}\left[t\right]\;|\;\rho \;=\;\frac{a}{100\%} \end{array}$$

where $v_{assisted}^{*}$, $v_{target}^{*}$, $v_{predicted}^{*}$ are normalized assisted, targeted, and predicted velocity vectors, respectively, and assistance ρ ∈ [0 … 1] was assigned with *a* ∈ [0… 100%] assistance level. That is for a zero level of assistance (ρ = 0) the displayed positions of the controlled virtual hand were calculated from the normalized predicted velocity vector without using the targeted velocity vector for correction.

The online part of the experiment with assisted feedback used 50% assistance level in the first run. The assistance level was decreased by 6% in each run resulting in 20% assistance level at the sixth run. Finally, the online part of the experiment providing the direct visual feedback used 0% assistance level. That is the subject performed the task without assistance for the third part of each session where the displayed position of the hand was calculated directly from the predicted velocity vector.

The target, predicted, and displayed kinematic data along with their corresponding EEG signals were stored for later evaluation of the online results. A general overview of the signal processing steps used for the online analysis is illustrated in the online section of **Figure 8**.

### Evaluation of the Offline and Online MTP Results

This section describes evaluation methods indicating the accuracy of the offline and online results. That is the accuracy of predicted and displayed virtual hand coordinate trajectories.

#### Methods to Prepare Figures Comparing Calculated and Target Trajectories

Here we describe the methods used for generating the figures that compare estimated and targeted trajectories of the virtual hands for the following five options:

■ **Predicted** trajectories from **offline** runs without visual feedback compared with targeted trajectories (Supplementary Figure 1)
■ **Predicted** trajectories from **online** runs using **assisted** visual feedback compared with targeted trajectories (Supplementary Figure 2)
■ **Predicted** trajectories from **online** runs using **direct** visual feedback compared with targeted trajectories (Supplementary Figure 3)
■ **Displayed** trajectories from **online** runs using **assisted** visual feedback compared with targeted trajectories (Supplementary Figure 4)
■ **Displayed** trajectories from **online** runs using **direct** visual feedback compared with targeted trajectories (Supplementary Figure 5).

It should be noted, that the predicted and displayed coordinates might be different as the displayed coordinates were limited to the area wherein the virtual arm was enabled to move (Figure 1) while as the coordinates of the predicted trajectories were not limited to the displayed area.

The sub-plots in Supplementary Figures 1–5 involve:

A comparison of estimated (predicted/displayed) and target trajectories using an averaged trajectory from 6-folds presented in subplot **(A)** of Supplementary Figures 1–5. The estimated trajectories for seven sessions, a cross-session average of estimated trajectories, and the targeted trajectory were plotted in the same sub-plot for each subject, hand, and Cartesian coordinates (i.e., x, y, z), separately. The comparison of estimated and targeted trajectories indicates how accurately an estimated (predicted and displayed) trajectory fits the target trajectory by comparing them in each of the three spatial dimensions, separately.

To compare the distance (error value) between the calculated hand position and each of the three targets (subplot **(B)** of Supplementary Figures 1–5), and home positions [subplot **(C)** of Supplementary Figures 1–5] the pairwise 3D distance between them was calculated for each subject and hand using a 3D distance metric presented in Box 1 and described in Equation (7):

(7) $$\begin{array}{r} d_{js}\left[t\right]=\sqrt{\overset{3}{\underset{i\;=1}{\sum }}{\left(x_{jsi}\left[t\right]-x_{jsi}^{\prime }\left[t\right]\right)}^{2}} \end{array}$$

where *d*_*js*_[*t*] is the 3D distance of *x*_*jsi*_[*t*] target and $x_{jsi}^{\prime }\left[t\right]$ is the predicted coordinates at time *t*. Subjects are indexed with *j*, sessions are indexed with *s*, and the spatial dimensions are indexed with *i*.

Results displayed in Supplementary Figures 1–5 were analyzed and compared by visual inspection.

#### Time-varying Decoding Accuracy of Predicted Trajectories

In order to calculate time-varying decoding accuracy (DA) for each subject and hand across all sessions and runs, trials belonging to the same subject and hand were pooled together from all sessions and runs. The distance between predicted and targeted hand coordinates was computed for each sample point. The classification of the predicted coordinates at a sample point within a trial was labeled as “success” if the 3D location designated by the predicted coordinates was closer to the actual target than to non-target locations for the trial. A ratio of the number of “successful” and “un-successful” classifications was calculated for each sample of each trial, session, run, hand and subject. Finally, the “success” classification ratio was converted into a percentage value and presented in the form of time-varying DA plots (**Figure 9**) indicating how the DA varied over time in the analyzed trials.

In order to validate the results obtained in the time-varying DA analysis, a permutation test was performed. For the permutation test, the order of the predicted trajectories associated with three different targets was randomly permuted for each subject and hand. During the trajectory randomization, 3D coordinates of the virtual arm (calculated by three separate MTP models) were associated and handled together. Time-varying DA plots generated from the original and randomly permuted datasets were displayed in the same sub-plot for each subject and hand, separately, and compared (**Figure 9**). Finally, the time-varying DA obtained using the original and randomly permuted datasets were compared using the Wilcoxon non-parametric test.

### Multi-class Classification Using Filter-Bank Common Spatial Patterns

The offline dataset was also assessed using a filter-bank common spatial patterns (FBCSP) (Ang et al., 2008) and mutual information (MI) selection (Pohjalainen et al., 2015) framework; a well-established multi-class classification technique used in BCI applications. The applied multi-class classification method enables discriminating between different types of imagined movements. Classification of imagined arm movements performed (A) with left vs. right arm and classification of imagined arm movements performed (B) with the same arm in three orthogonal directions provides information to enable the control of two virtual arms in 3D. Furthermore, we wanted to know (C) whether any differences could be detected in motor imagery tasks performed during sub-blocks 1 vs. sub-block2. Thus, we investigated, with an FBCSP method could accurately classify imagined arm movements performed (A) with different arms, (B) with the same arm in three orthogonal directions, and (C) in sub-blocks 1 vs. sub-block2 (Table 1).

**Table 1:** Investigated class setups for FBCSP-based multi-class classification.

**Analysis ID**	**Class number**	**Investigated classes**
A	2-class classification	Motor imagery of the left vs. right arms (Independently from the movement direction)
B	3-class classification	Imagined movement of the left hand toward three targets (Left X, Left Y, Left Z)
		Imagined movement of the right hand toward three targets (Right X, Right Y, Right Z)
C	2-class classification	Task performance in sub-blocks 1 vs. sub-block2 (Handling together all tasks in both sub-blocks independently from task types)

**Figure 7:**
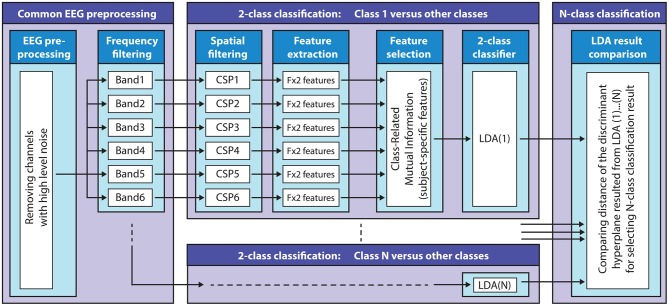
Filter-bank common spatial patterns (FBCSP) based multi-class classification method. The block diagram illustrates the structure of the FBCSP based multi-class classification method using a mutual information (MI) selection module and linear discriminant analysis (LDA) based classifier.

The FBCSP based multi-class classification method used in this study was described in a recent study aimed at classifying mental imagery of five primitive shapes (Korik et al., 2018a). A general overview of the applied FBCSP method is presented in Figure 7.

#### Pre-processing

***Frequency filtering***: signals were band-pass filtered in six non-overlapped standard EEG bands [0.5–4 Hz (delta), 4–8 Hz (theta), 8–12 Hz (mu), 12–18 Hz (low beta), 18–28 Hz (high beta), and 28–40 Hz (low gamma)] with Simulink using high-pass and low-pass FIR filter modules (band-pass attenuation 0 dB, band-stop attenuation 60 dB).

#### Multi-class Classification Using FBCSP

The multi-class classification module involves multiple two-class classifiers (target vs. non-target classes) to separate each target class from the other (non-target) classes. Thus, the number of the two-class classifiers equaled the number of the classes. The FBCSP method (subject-specific temporal-spatial filter) was applied to each 2-class classifier using the six band-pass filtered EEG channels to perform an autonomous selection of key temporal-spatial discriminative EEG characteristics. ***Spatial filtering:***a CSP module was applied to each 2-class classifier as a feature extraction method to create spatial filters to maximize the discriminability of two classes by maximizing the class conditional variance ratios (Lotte and Guan, 2010, 2011).

The number of the CSP filter pairs for each 2-class classifier for each subject and frequency band was reduced and optimized based on the obtained CSP discriminative filter pairs (described above). ***Feature extraction:***the time-varying log-variance of the CSP filtered EEG was calculated using a 1 s width sliding window with a 200 ms time lag between two windows. The offset of the sliding window was set to cover the time interval between −4 s (prior) and 12 s (after) the onset of the imagined movement. This interval included a full imagined movement cycle (presented in Figure 2A). ***Feature selection:***the mutual information (MI) between features and associated target class using a quantized feature space was estimated (Pohjalainen et al., 2015) to identify features that can discriminate a target class from other (non-target) classes. ***2-class classification:***A regularized LDA (RLDA) algorithm [from the RCSP toolbox (Lotte and Guan, 2010)] was applied to classify the extracted features to the actual target or non-target class. ***Multi-class classification:***the final DA for multi-class classification was decided by assessing the signed distance in the 2-class discriminant hyper-plane for each target vs. non-target related binary classifiers.

#### Optimal Parameter Selection, Decoding Accuracy, and Cross-Validation

The optimal parameter selection and multi-class DA calculation were processed in the framework of the inner-outer (nested) cross-validation (CV). The inner-outer CV enables testing and selecting a range of parameters using an inner fold CV and calculating the final test results in the outer fold CV using the optimal architecture selected by the inner fold CV. For both levels in the inner-outer CV (i.e., for inner and outer level CVs), a leave-out-one CV was applied (described in Figure 6). A dataset that was used for training at the outer level leave-out-one CV was spilt into test and training fold configurations during the inner level leave-out-one CV. This data separation guarantees that the test data of the outer level CV would not be used in the inner level for parameter optimization.

For single session analysis (using a dataset recorded from a single session), six outer folds were assigned (matching the six runs) and five inner folds were assigned. For multi-session analysis (using datasets from seven sessions of the same subject), the number of the outer folds was set to seven (matching the number of the sessions) and the number of the inner folds was set to six. During the inner fold CV the optimal architecture selection denoted the number of the selected CSP filter pairs, the number of the quantization levels for MI processing module, and the number of the selected feature at the output of the MI module. Further details of the inner-outer CV is described in Korik et al. (2016a).

The Wilcoxon non-parametric test was used to validate the difference of DA prior the task performance pause/resting period (−1 s) and at the maximal peak accuracy occurring during the motor imagery task (0–10 s) is significant (*p* < 0.01).

The analysis was carried out using a dataset comprising trials from one of the two sub-blocks, separately. First sub-block: the task was performed during visual and audio stimuli (i.e., the virtual arm displayed the movement during task performance). Second sub-block: the task was performed during audio cue without displaying the movement (i.e., the virtual arms did not leave the home position during the task performance). Furthermore, an additional analysis was performed using a dataset involving trials from both sub-blocks. The time-varying DA was calculated and plotted from final test results (outer test folds) based on single-session and multi-session analysis.

In order to identify frequency bands and cortical areas that provide the highest contribution for DA, an analysis was performed using a multi-session dataset involving CSP filters and MI weights of the FBCSP classifiers trained for each subject, session, and outer fold, separately. For the frequency analysis, the mean values of MI weights (used for weighting features of the 2-class classifiers) were calculated in each analyzed frequency band, separately. The obtained results were plotted in form of subject-specific heat maps indicating time-varying DA contribution of the analyzed frequency bands. For the topographical analysis, the averaged pattern of the MI weighted CSP filters (used for generating input for feature extraction prior the MI and 2-class classifier modules) were calculated in each analyzed frequency band at the time point that provided maximal DA. The cross-subject average of the MI weighted CSP filters was plotted in form of cross-subject topographical map for each analyzed frequency band, separately, indicating DA contribution of different cortical areas in the relevant frequency band.

A summary of the analyzed class setups options is presented in Table 1.

### Methods Summary

**Figure 8:**
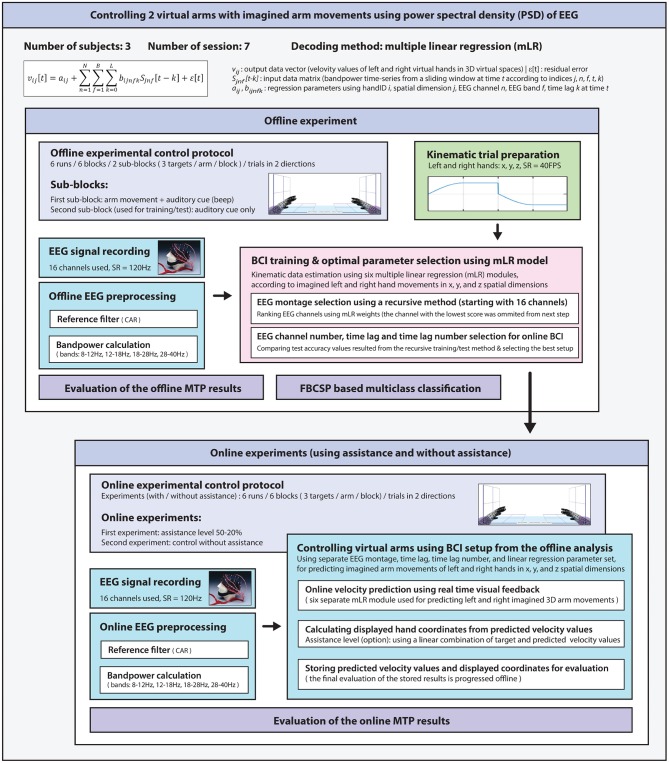
A general overview of the methods used for this study.

A general overview of the methods used in this study is presented in Figure 8.

## Results

### MTP Results From the Offline and Online Parts of the Sessions

A visual inspection of the results presented in Supplementary Figures 1A1–A3 for most sessions showed that the speed of the imagined identical (non-periodic) movements was estimated correctly by the applied MTP model in the direction that matched the direction of the imagined movement. However, visual inspection of the results also showed that the predicted velocity vector in 3D space had a significant error resulting from incorrectly predicted velocity vector components in the non-target directions (i.e., in directions that did not match the direction of the imagined movement). Furthermore, a high level of baseline shift of the predicted velocity vectors [detected in form of linear shift of the predicted coordinates in most of the sub-plots (A1–A3) of Supplementary Figures 2, 3] had a negative impact on the online accuracy rate as it resulted in a constant velocity component of the virtual arm movement during online task performance.

#### Time-varying DA Plots

**Figure 9:**
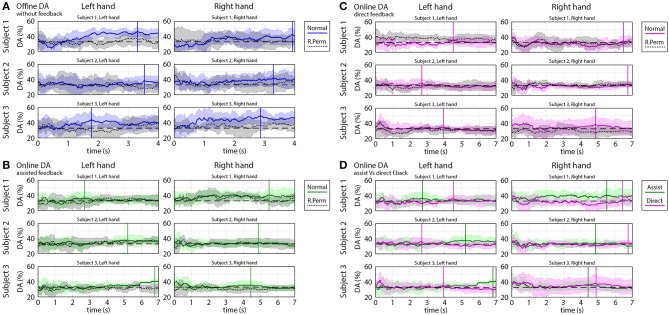
Time-varying decoding accuracy (DA) of predicted trajectories in offline and online parts of the experiments. The time-varying DA plots show how varying the ratio of the “successful” and “un-successful” target classifications over time during a movement cycle from the home position to a target position. DA values were calculated based on predicted coordinates of the virtual hand as described in the Methods section. **(A)** Offline DA without feedback. **(B)** Online DA with assisted feedback. **(C)** Online DA with direct feedback. **(D)** Comparison of online DA using assisted vs. direct feedback. **(A–D)** present the mean value (colored line) and standard deviation (colored shaded area) of the time-varying DA and **(A–C)** present also the results of the permutation test (mean: black dotted line, standard deviation: gray shaded area). DA peaks obtained during task periods are indicated in **(A–D)** with vertical solid lines. The chance level (33.3% for 3-class classification) is denoted by a black dashed line.

Figure 9 shows time-varying DA of predicted trajectories from offline and online. Subjects 1 and 3 in most of the sessions achieved significantly higher peak accuracy (DA^peak^ mean 45%, std 5%) in the offline part of the experiments (Figure 9A) and in the online part of the experiments with assisted visual feedback (DA^peak^ mean 40%, std 5%) (Figure 9B) than chance level (33.3% for 3-class classification). However, a significant difference between DA calculated from the original dataset and randomly permuted dataset (Wilcoxon non-parametric test, *p* < 0.05) was found only for the offline results.

The DA in the online part of the experiments in which the subjects received a direct visual feedback (without assistance) was in the range of 33% chance level (DA^peak^ mean 35%, std 5%) (Figure 9C) and DA calculated from the original, and randomly permuted dataset were similar, i.e., a significant difference was not found (Wilcoxon non-parametric test, *p* > 0.05).

During task performance, the peak accuracy of predicted hand coordinates for online part of the experiments for trials with assisted visual feedback was, in most of the cases, significantly higher (Wilcoxon non-parametric test: *p* < 0.05) than for trials with no direct visual feedback assistance (20–50% correction in displayed coordinates) (Figure 9D).

It has to be noted that decoding accuracy for online assisted experiments was calculated from the predicted kinematic parameters and not from the displayed coordinates, which were partially fitted to the target trajectory using a ratio designated by the assistance level.

### FBCSP Based Multi-class Classification Results From the Offline Runs

This sub-section presents the results of the offline runs using an FBCSP based multi-class classification method.

#### Classification of Imagined Movements Performed With the Left or Right Hand

**Figure 10:**
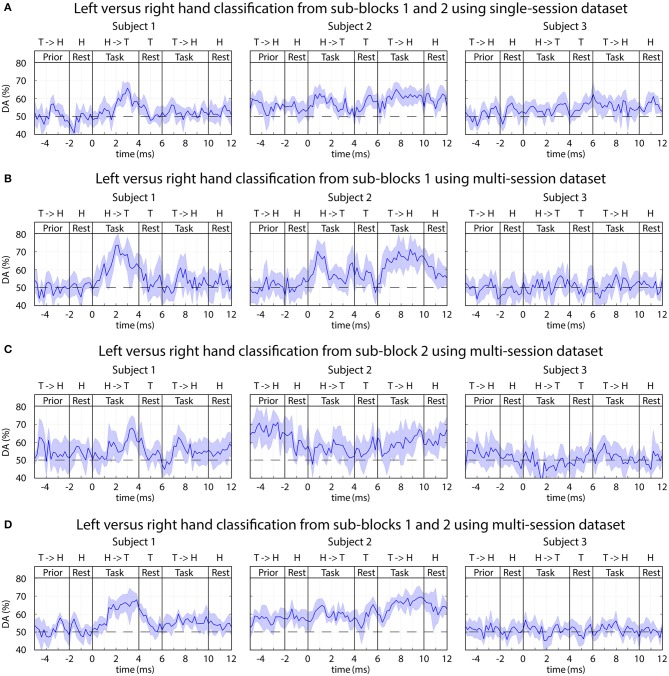
FBCSP: time-varying DA of Left vs. right imagined hand movements. **(A)** Results of the single-session analysis. **(B–D)** Results from the multi-session analysis where **(B)** trials from sub-block1 (visual and auditory cue), **(C)** trials from sub-block2 (auditory cue without displayed movement), **(D)** trials from both sub-blocks pooled together. The horizontal dashed line indicates the chance level (50% for 2-class classification).

The time-varying DA of 2-class classification separating imagined movements of the two arms is presented in Figure 10, and peak accuracy rates achieved for separating imagined movements of the two arms are summarized in Table 2.

**Table 2:** Peak accuracy achieved for separating imagined movements of the left and right arm.

**Subject ID**	**Multi-session analysis (the classifier was trained using trials from all sessions)**	**Multiple single-session analysis (the classifier was trained using trials from only one session)**
Subject 1	DA^peak^ = 75%	DA^peak^ = 65%
Subject 2	DA^peak^ = 70%	DA^peak^ = 63%
Subject 3	DA^peak^ ≈ chance level (50%)	DA^peak^ ≈ chance level (50%)

Subject 1 (who was previously trained to control a BCI using left vs. right motor imagery paradigm) and Subject 2 (a naïve subject) in both of the multi-session and single-session analyses achieved a reasonable level of accuracy (DA^peak^ mean 70%, std 5%) for separating imagined movement of the left and right arms. The peak accuracy for subjects 1 and 2 was significantly higher (*p* < 0.001, Wilcoxon non-parametric test) than DA obtained from 0 to 2 s prior to the onset of the tasks (which was equal to the chance level of 50% for 2-class classification). For subject 3 (a naïve subject), the left and right tasks were not separable (DA ≈ 50% for both single-session and multi-session comparisons).

#### Classification of Single Arm Movements Toward Three Different Targets

In order to discriminate between imagined arm movements performed with the same arm toward the three corresponding targets, an analysis was performed with the FBCSP based classification method using one of the following three datasets.

1. Trials from sub-block 1, during displayed movement
2. Trials from sub-block 2, during non-displayed movement
3. Trials collected from both task sub-blocks.

**Figure 11:**
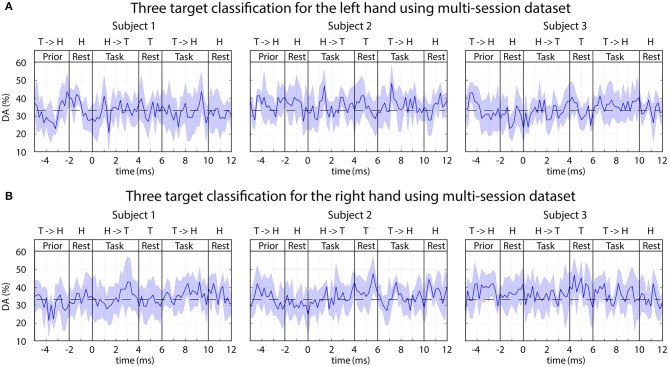
FBCSP: time-varying DA during imagined movements toward three different targets. The 3-class DA presented in this figure was calculated from a multi-session dataset based on the imagined movement of the left **(A)** and right **(B)** hands using sub-blocks without displaying movement on the screen (auditory stimulus only). The horizontal dashed line indicates the chance level (33% for 3-class classification).

Each of the above-presented options was analyzed using a single-session and multi-session based training but none of them provided DA significantly different from the chance level (33% for 3-class classification). An example of the obtained time-varying DA from multi-session analysis using trials recorded during auditory stimuli without displayed movement is presented in Figure 11.

#### Classification of EEG in Sub-block 1 vs. Sub-block 2

**Figure 12:**
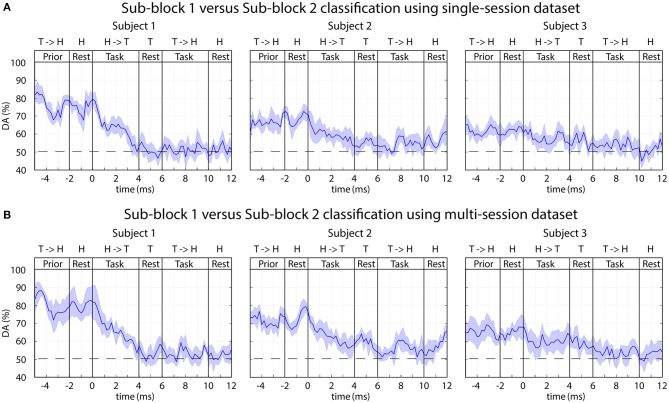
FBCSP: time-varying DA comparing task performance in sub-blocks 1 vs. sub-block 2. The 2-class DA presented in this figure was calculated from single-session **(A)** and multi-session **(B)** based datasets comparing sub-blocks with (sub-block 1) and without (sub-block 2) displaying the expected movements. The horizontal dashed lined indicates the chance level (50% for 2-class classification).

The time-varying DA results from an analysis, separating task performance in two sub-blocks is presented in Figure 12.

A reasonably high level of peak accuracy (DA^peak^ ≈ 80%) was achieved prior the onset of the imagined movements (between −5 and 0 s) for separating sub-block 1 and sub-block 2 and the accuracy has decreased to the chance level (50% for 2-class classification) after the onset of the task performance (0 s) (Figure 12). It is important to note, sub-blocks 1 and 2 were not separated with an inter-sub-block resting period. Thus, the time interval where the peak accuracy achieved for sub-block 1 it is matched the resting period prior sub-block 1; for sub-block 2 it is matched the end of the task period in sub-block 1 prior the onset of sub-block 2 (Figure 2). Therefore, the peak accuracy is achieved when a task initialization period (prior the onset of sub-block 1) was compared with a task period (prior the onset of sub-block 2).

**Figure 13:**
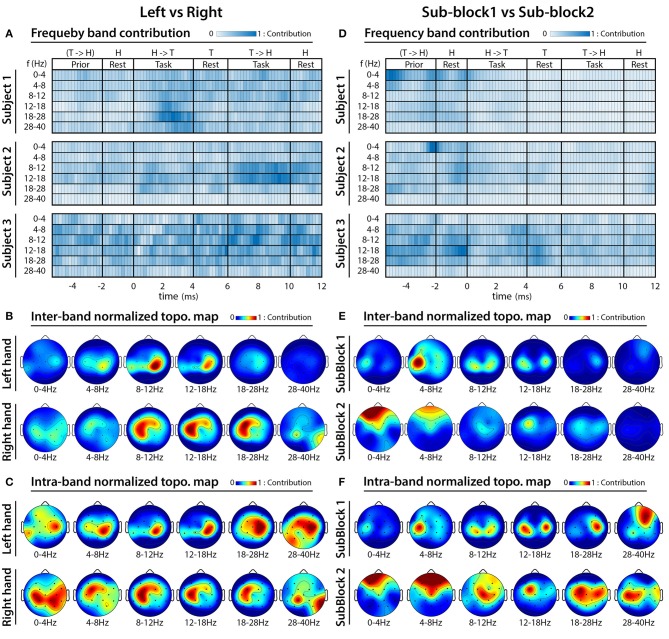
FBCSP: results of frequency analysis and topographical maps using multi-session dataset. **(A–C)** Indicate which frequency bands and cortical areas provided the highest contribution for separating imagined movements performed with left vs. right hands at that time for which the classification accuracy was maximal. **(D–F)** Indicate which frequency bands and cortical areas provided the highest contribution for separating imagined movements performed in sub-block 1 vs. sub-block 2 at that that time for which the classification accuracy was maximal. The subject-specific frequency band contribution maps **(A,D)** were calculated at peak accuracy using MI weights of the trained 2-class classifiers, which were applied to the multi-class classifier module. The cross-subject topographical maps **(B,C,E,F)** were calculated using average of the MI weighted CSP filters, which were used from subject-specifically trained 2-class classifiers applied to the multi-class classifier module. For calculating an averaged pattern for the cross-subject topographical plots, the MI weighted CSP filters subject-specifically were selected at peak accuracy of the corresponding subjects.

Figures 13A–C presents results of the analysis performed to identify frequency bands and cortical areas that allow the best separation of imagined movements performed with the left and right hand; and Figures 13D–F presents that of separating tasks performed in sub-block 1 and sub-block 2. As the FBCSP method was not success in separation of imagined movements performed toward different directions with the same hand, the frequency and topographical analysis for movement direction classification was omitted.

## Discussion

Decoding the trajectory of imagined movements from EEG has been reported in a limited number of studies and to the best of the authors' knowledge, none of these studies provided real-time feedback to the subject about task performance for close loop control. Many motion trajectory prediction/decoding focus on executed movement and do not consider imagined movement.

Closely related studies regarding trajectory prediction of imagined arm movements include Kim et al. (2015) who decoded 3D trajectory of executed and imagined arm movements performed in parallel with the observation of a human volunteer or robot performing 3D arm movements. Decoded trajectories in Kim et al. (2015) were calculated using two different methods: a multiple linear regression (mLR) method and a kernel ridge regression (KRR) method, and compared. The correlation coefficient of target and predicted trajectories using multiple linear regression resulted in R ≈ 0.35 for executed movements, and R ≈ 0.3 for observed and imagined movements; based on the kernel ridge regression these values are resulted in R ≈ 0.5 for executed movements, and R≈0.4 for observed and imagined movements.

Ofner and Müller-Putz (2015) decoded motor imagery tasks involving imagery of arm movement in vertical and horizontal directions of a 2D plane synchronized with a metronome (DA mean 64%, std 11% compared to 50% chance level). However, both studies (Kim et al., 2015; Ofner and Müller-Putz, 2015) used an open loop scenario and the analysis in these studies was performed using an offline recorded dataset. Müller-Putz et al. (2018) presented two closely related studies to classify in closed-loop six natural joint movements of the same arm and three different grasp types from motor-related cortical potentials (MRCPs) in a narrow 0.3 to 3 Hz band. For the first study, the achieved classification accuracy was 37% (chance level was 16.7% for six-class classification) and the second study showed grasps possible to decode from MRCPs features (binary classification of 74% grasp vs. 100% grasp).

Other studies, reviewed in the introduction section (Wolpaw and McFarland, 2004; Lehtonen et al., 2008; Fukuma et al., 2015; Bhagat et al., 2016; Hotson et al., 2016; Lee et al., 2017; Müller-Putz et al., 2018), aimed to achieve online control of objects in 2D/3D real and virtual spaces, however, none of these studies provided real-time control of an artificial, robotic, or virtual arm using the 3D trajectory of imagined arm movements decoded from EEG.

Our present study aimed to investigate whether the 3D trajectory of imagined hand movements could be decoded online in real-time from EEG in closed-loop where two virtual 3D arms are presented on a 2D screen. This scenario or experiment has not previously been attempted.

The seven-session experiment, comprising each an offline and two online experimental modules, was completed by three subjects. The parameters of the BCI were tuned using datasets recorded during the offline runs, and the trained BCI was used to control the two virtual arms in the online runs. The major step here is the attempt to decode in real-time imagined arm movements, as opposed to offline decoding reported in our previous works (Korik et al., 2015, 2016a,b,c).

### Evaluation of the MTP Results

The present study showed that decoding the 3D trajectories of imagined arm movements toward three targets per arm is a challenging objective. Although, the accuracy rate achieved in offline runs for Subjects 1 and 3 (DA^peak^ mean 45%, std 5%) was significantly higher (*p* < 0.05) than the chance level (33.3% for 3-class classification) (Figure 9A), the decoding accuracy in online runs fell below 40%.

A possible reason for this difference might be that, before the online runs, the subjects were asked to control the virtual arm by correcting the position whilst observing the visual feedback (i.e., always attempting to move the arm toward a direction of the actual target or home position depending on the actual arm position displayed on the screen). Thus, the subjects may performed different motion imagery strategies during the offline and online runs of the sessions. Furthermore, the online part of the experiments used a BCI that was trained using a dataset recorded in offline runs. Thus, the strategies used by the subjects to generate imagined arm movements during online runs presenting direct or assisted feedback may have differed from that of used in the offline runs.

An additional putative cause for the enhanced accuracy rate in online runs with assistant visual feedback might be that during most online sessions, a significant baseline shift of the predicted velocity vectors was detected. As the virtual hand coordinates were calculated by integrating velocity vectors during a movement trial, the error in predicted velocity vectors resulted in a constant velocity component of the virtual hand movement (sub-plots A1-A3 of Supplementary Figures 2, 3). This constant translation of predicted coordinates of the virtual hand led to an over-balanced negative visual feedback during online control without assistance. However, the ratio of the negative and positive feedbacks was more balanced when an assisted visual feedback was provided. Thus, the difference of the accuracy rates achieved in online runs using assisted vs. direct visual feedback (Figure 9D) might originate from the enhanced negative feedback.

This explanation is in line with studies showing that negative feedback has a significant impact on accuracy during online task performance. In Huang and Yu (2014), a component of event-related EEG potential called feedback-related negativity (FRN) was found to be sensitive to negative feedback as well as to a negative prediction error. Moreover, Alimardani et al. (2014) highlighted that a biased feedback is an important component in motor imagery BCI systems. They used an EEG based BCI-operated human-like robotic hands to study subjects' performance under different presentations of the feedback (non-biased direct feedback, biased feedback corrected to fake positive 90% accuracy, or fake negative 20% accuracy ratio) using imagine a grasp or squeeze motion. They found that, subjects achieved a better accuracy rate when they received fake positive feedback while fake negative feedback resulted in a decrease in the classification accuracy. Our results are in line with Alimardani et al. (2014) and suggest that, an un-balanced highly negative real-time feedback, which is not ignored by the subject, may have a negative impact on the subject's performance. Furthermore, the results also indicate that, a proper balance between the real-time positive and negative feedbacks provided to the BCI user during an online multi-session learning period is an important factor that may help achieve a reasonably high accuracy rate in experiments using a closed-loop scenario.

### Evaluation of the FBCSP Based Multi-Class Classification Results

This section discusses the FBCSP based multi-class classification results.

#### Classification of Imagined Movements Performed With Left vs. Right Hands

The results showed that using a dataset from whom the classifier was trained using trials from multiple sessions achieved higher DA (Figure 10D Subjects 1 and 2, DA^peak^ mean 70%, std 5%) compared to the average accuracy of multiple single session based classification (Figure 10A Subject 1 DA^peak^ mean 65%, std 5%, Subject 2 DA^peak^ mean 63%, std 5%). The difference in single-session vs. multi-session based analysis may originate from the difference in the number of trials used for training the classifier. In the single-session based training the number of trials was low for both of the following options: 5 trials/class using trials from one of the two sub-blocks, or 10 trials/class using trials from both sub-blocks. However, the number of trials for the multi-session based training was seven-times more resulting in 35 trials/class using trials from one of the two sub-blocks, or 70 trials/class using trials from both sub-blocks. The low number of trials is sub-optimal for the applied FBCSP classification method, but it is should be mentioned FBCSP multi-class classification method was not part of the planned analysis when the experimental paradigm was designed.

The frequency and topographical analysis indicated that the task related EEG activity in 8–12 Hz (mu) and 12–28 Hz (beta) bands in the contralateral sensorimotor cortex around C3 and C4 electrode locations provided the highest contribution for separating imagined movements of the left and right hand (Figures 13A–C). This observation is in line with the literature (McFarland et al., 2000) reporting the highest classification accuracy for separating imagined movements of left and right hand using the power spectral density (PSD) of mu (8–12 Hz) and beta (12–28 Hz) bands in the contralateral sensorimotor cortex.

#### Classification of Single Arm Movements Toward Three Different Targets

Although the applied FBCSP method provided reasonable accuracy for left vs. right hand movement classification (Figure 10), classification of single arm movements toward three different targets using the same dataset did not result in a reasonable accuracy rate for any of the three subjects (Figure 11). In contrast to FBCSP results, MTP method applied in this study using the same multi-session offline dataset for subjects 1 and 3 achieved a DA (in classification of predicted imagined movement trajectories toward three targets/hand) that was significantly higher than chance level (DA^peak^ mean 45%, std 5%) (Figure 9A). The difference may originate from different numbers of feature vectors used for training the FBCSP based multi-class classifier and MTP methods.

- The FBCSP based multi-class classifier was trained using a dataset involving only one feature vector per trial. Furthermore, features within the feature vector that was used for training the classifier were calculated from a single time-window (specified by width of the window and offset of the window compared to onset of the task), therefore, the number of the feature vectors used in training of the FBCSP classifier was only 7 sessions × 5 runs × 1 trial/target = 35 trials/target –> 35 feature vector/class.
- In contrast to the applied FBCSP based classifier, the applied MTP model uses multiple feature vectors per trial for training the kinematic data estimation module. Similarly to the FBCSP classifier, each feature vector in the MTP model comprised a specific number of features selected using a feature window. In contrast to the FBCSP classifier, the MTP model does not only use one feature vector per trial for training the MTP model but uses a sliding feature window for collecting multiple feature vectors. Thus, the MTP method provides a bigger pool of input data for training the MTP module than the applied FBCSP method selecting a single feature vector per trial.

A combination of the two above-mentioned methods (i.e., selecting multiple feature vectors per trial for training the classifier) might improve the accuracy of the classification by taking advantage of each of the models. It should be tested in a future work.

#### Classification of EEG in Sub-block 1 vs. Sub-block 2

Several seconds (−5–0 s) prior to the onset of the motor imagery task, a reasonable high level of peak accuracy (DA^peak^ ≈ 80%) was obtained for classification of EEG signals recorded in sub-block 1 and sub-block 2. This time interval for sub-block 1 matched a resting period prior the onset of sub-block 1 and for sub-block 2 this time interval involved task performance in sub-block 2 prior the onset of sub-block 2 (Figure 2). Thus, the achieved peak accuracy probably originated from classification of task vs. resting conditions and did not originate from classification of different task performance within the two sub-blocks. This statement is supported by the finding that several seconds before the onset of the task the DA was maximal, and after the onset of the tasks (when in both sub-blocks the motor imagery task performed), the DA fell significantly.

The frequency analysis indicated that subjects specific frequency bands [for subject 1 and 2 the 0–4 Hz (delta) band, for subject 3 the 8–12 Hz (mu), and 12–28 Hz (beta) bands] provided the highest contribution for separating EEG signals recorded prior the onset of the task performance in sub-blocks 1 and 2 (Figures 6, 13A). Furthermore, the topographical analysis indicated that in the most frequency bands the sensorimotor cortex around C3 and C4 electrode positions provided the highest contribution to the classification accuracy. However, in the 0–4 Hz (delta), 4–8 Hz (theta), and 28–40 Hz (low gamma) bands the frontal areas also provided a reasonable contribution. The results support the notion that delta, mu, and beta EEG oscillations in the sensorimotor cortex comprise information that may enable to separate motor imagery tasks from a time interval related to a resting period prior task performance.

### Limitations and Proposed Modifications

The experimental paradigm that was used in this study was designed based on the paradigm applied in our previous study wherein 3D trajectory of imagined arm movements were decoded offline from EEG (Korik et al., 2018b). However, in order to close the control loop and allow online decoding of the imagined trajectories, some key elements of the previous paradigm were re-examined and modified. This section summarizes the limitations and issues associated with the approach applied, and presents a proposed modification of the paradigm using experiences gained from the present work.

#### The Critical Issues of the Present Paradigm

- Prior to commencing the online part of the experiments, the subjects were asked to control the virtual arm during the online tasks using the presented visual feedback. This instruction may have resulted in different control strategies for virtual hand movement between the offline runs (when the BCI was trained) and online runs (when the subject used the BCI). Thus, subjects in offline and online sessions might have produced significantly different EEG patterns for an imagined movement between the home position and a specific target position. Therefore, ignoring the feedback during the time-period in which the subject learns to use the BCI might lead to higher accuracy.
- The present study trained subjects to control two virtual arms, rather than one virtual arm, by motor imagery. This setup increased the complexity of the experimental paradigm and reduced the amount of trials per hand.
- As the EEG system differed from our earlier studies (data recorded at HIT, Israel) we modified the montage, firstly to simplify the setup with less electrodes and including only a single reference electrode. This meant that we could not apply directly the same approach as in Korik et al. (2018b). As there were a number of significant changes between the current online study and the offline study comparison of results across studies is limited. In hindsight minimization of differences between studies would have been more informative.
- The number of trials per hand and target in the offline session was only six. This number is significantly lower than the number of trials (80 per target) used in our previous study (Korik et al., 2018b). We expected long trials (4 s for each direction in the offline sessions) would compensate for this drawback as feature vectors for training the BCI would be collected over a long period during the imagined movement trials. This modification in the paradigm may be sub-optimal resulting in an improper training of the BCI, therefore, in the future work the current paradigm should be modified. The duration of the trials should be shorter (1 s instead of 4 s) along with the pause periods (1 s instead of 2 s). Sub-blocks with displayed movements should not be involved in the offline paradigm, i.e., to keep only sub-blocks without displaying movements. Furthermore, the block initialization time should be shorter by using visual cues (0.5–1 s) instead of audio messages (2 s) informing the subject about the next target. The above listed modifications should enable recording a significantly higher number of trials during the same time-period and could lead to a more optimal training of the BCI.
- The BCI in each session was re-trained for the online part of the experiment using a dataset recorded at the beginning of the session during an offline runs. Thus, the input feature space selected for the online part of the experiment (involving the optimal EEG montage, time lag, and number of the time lags) varied between each session. This strategy did not support a consequent learning process for the subject across multiple sessions, which would require using a more stable input feature space. (Lin et al., 2017) presented a minimization of single-session EEG variability of emotional responses using an attempt to facilitate cross-session emotion classification. A multi-session analysis based feature selection method comparing stability of DA using different groups of features could lead to better BCI performance during a multi-session online experiment series.

The accuracy metrics introduced in this study (Box 1) measures the 3D distance of predicted and expected coordinates in the form of a single scalar value as a non-linear combination of error rates from three separately trained MTP models (each linked to one of the three orthogonal basis vectors of the 3D virtual spaces). The accuracy metrics were used to attach the three separate MTP models for selecting an optimal value for some structural model parameters (EEG montage, time lag and time lag number) and the same accuracy metrics used for result evaluation. However, the three separate linear regression models [v(x), v(y), and v(z)] were kept un-attached during training of the internal model parameters (i.e., linear regression parameters) because of the limitation of the applied mLR training method.

In the future works, the number of subjects should be increased and the experimental paradigm should be revised according to the critical issue discussed above. Also, it would be advantageous to study subjects over more sessions and to increase the number of trials per session to investigate performance improvement and learning in a closed-loop scenario.

## Conclusion

To date, only a limited number of studies reported decoding the trajectory of imagined limb movements from EEG, and to the best of the authors' knowledge, the real-time decoding of imagined 3D arm movement trajectories from EEG has not been studied yet in a closed-loop. In this research, we aimed to address this gap by studying real-time control of two virtual arms toward three corresponding targets placed at orthogonal angles from the viewpoint of the home position of each hand. The virtual arms, displayed on 2D screen, were controlled by an online BCI that attempted to decode the 3D trajectory of imagined arm movements with multiple linear regression and power spectral density of mu, low beta, high beta, and low gamma oscillations. The BCI was trained using an offline EEG dataset recorded at the beginning of each session before the online runs providing assisted and direct visual feedback in a closed-loop using predicted coordinates of the virtual arm. Although for two of three subjects a reasonably high accuracy of imagined 3D trajectory prediction for offline task performance was achieved (DA^peak^ mean 45%, std 5%, chance level 33.3%), the accuracy during real-time control of the virtual arms using the trajectory decoded directly from EEG for all subjects was in the range of chance level (33.3%). Online performance of the two subjects with highest accuracy in offline runs shows, that false-positive feedback may have a positive impact on the accuracy using a closed-loop scenario as suggested in Alimardani et al. (2014). The difference in accuracy between online runs involving assisted vs. direct (non-assisted) visual feedback highlights that an overbalanced negative biofeedback can lead to a negative impact on the accuracy.

To compare the accuracy of the imagined arm movement trajectory prediction (MTP) method with the performance of filter-bank common spatial patterns (FBCSP) and mutual information (MI) based multi-class classification method, the time-varying decoding accuracy was calculated for both methods using the offline datasets and compared. Using the current experimental paradigm, the FBCSP-MI based classifier separated left and right hand movements with reasonably high accuracy (DA^peak^ mean 70%, std 5%, chance level 50%). However, classification of the imagined movement toward different targets with the same arm was successful only with the MTP. The significant difference in achieved accuracy using MTP methods vs. FBCSP-MI classifier for target classification might originate from the low number of trials provided by the present experimental paradigm. As the applied FBCSP-MI classifier used only one feature vector per trial to train the classifier for separating a certain number of classes, the low number of training trials was a critical issue for the FBCSP-MI method.

The results of this pilot study provide an encouraging step forward in the research and development of paradigms for BCIs targeting physically impaired individuals to perform movement-independent communication and online control in real and virtual spaces. However, as highlighted in the discussion section we recommend a range of adaptions to the paradigm for future studies.

## Ethics Statement

Prior to the research beginning, subjects were presented with information about the experimental protocol and asked to read and sign an informed consent form to participate in the study, which was approved by the Ulster University research ethics committee (UREC).

## Author Contributions

AK carried out the research, data collection, computation and data analysis, and prepared the figures and draft of the manuscript. RS reviewed and revised the manuscript. NS supervised the research and revised the manuscript. DC supervised the research, contributed to experimental design, data and results verification, literature search and discussion of the results, and revised the manuscript.

### Conflict of Interest

The authors declare that the research was conducted in the absence of any commercial or financial relationships that could be construed as a potential conflict of interest.
