# Supplementary material for: Decoding Imagined 3D Arm Movement Trajectories From EEG to Control Two Virtual Arms—A Pilot Study
Source: Front Neurorobot. 2019 Nov 14;13:94. doi: 10.3389/fnbot.2019.00094 (PMC6868122; doi:10.3389/fnbot.2019.00094)
Supplement: Supplementary file 1 [file Data_Sheet_1.PDF]

Supplementary Material

Decoding Imagined 3D Arm Movement Trajectories from EEG to Control Two Virtual Arms - A Pilot Study

Attila Korik*, Ronen Sosnik, Nazmul Siddique, Damien Coyle

*** Correspondence:** Corresponding Author: a.korik@ulster.ac.uk

Comparison of calculated and target trajectories

Subplots **(A1-A3)** of **Supplementary Figure 1** indicates for the **offline runs,** velocity vector component (x, y, or z) which matches the direction of the actual movement [i.e., those trials in which the targeted coordinates (thick black dotted line) pick up non-constant (zero) values]. As it is shown, the orientation (home-to-target or target-to-home) of the predicted movements (thick black solid line) in the most session match the orientation of the target movement (thick black dotted line) indicating the direction of the predicted movement is the same as the targeted direction. However, for velocity vector components, which oriented orthogonally to the direction of the imagined movement [i.e., do not match the direction of the actual movement], the value of the velocity vector component commonly picks up a (non-zero) value which scaled in a similar range as the speed of the targeted movement.

The results for most sessions show, the speed of the imagined identical (non-periodic) movements is estimated correctly by the applied MTP model in the direction which matches the direction of the imagined movement. However, the predicted velocity vector in 3D spaces has a significant error resulting from incorrectly predicted velocity vector components in the non-target directions.

Subplots **(A1-A3)** of **Supplementary Figures 2 and 3** indicate for the **online runs** (using **assisted and direct visual feedback**, respectively) that, the **predicted** velocity vectors involve relatively high level of baseline shift. The baseline shift of the predicted velocity vectors (detected in form of linear shift of the predicted coordinates in most of the sub-plots **(A1-A3)** of **Supplementary Figures 2 and 3**) caused a critical issue for the online part of the experiments as it induced a constant translation of the virtual arm during online task performance. The **displayed** coordinates of the virtual hand from **online runs** using **assisted and direct visual feedback** are presented in subplots **(A1-A3)** of **Supplementary Figures 4 and 5**, respectively.

The time-varying distance between the 3D location of the calculated hand position and each of the three targets are plotted in subplot **(B)** of **Supplementary Figures 1-5**. Distance values presented in subplot **(B)** of **Supplementary Figures 1-3** were used for preparing time-varying DA plots (see Figure 6.9 in the main text body) for offline and online parts of the experiments).

The time-varying distance between the 3D location of the calculated hand position and actual home position is presented in (subplot **(C)** of Supplementary Figures 1-5**).**

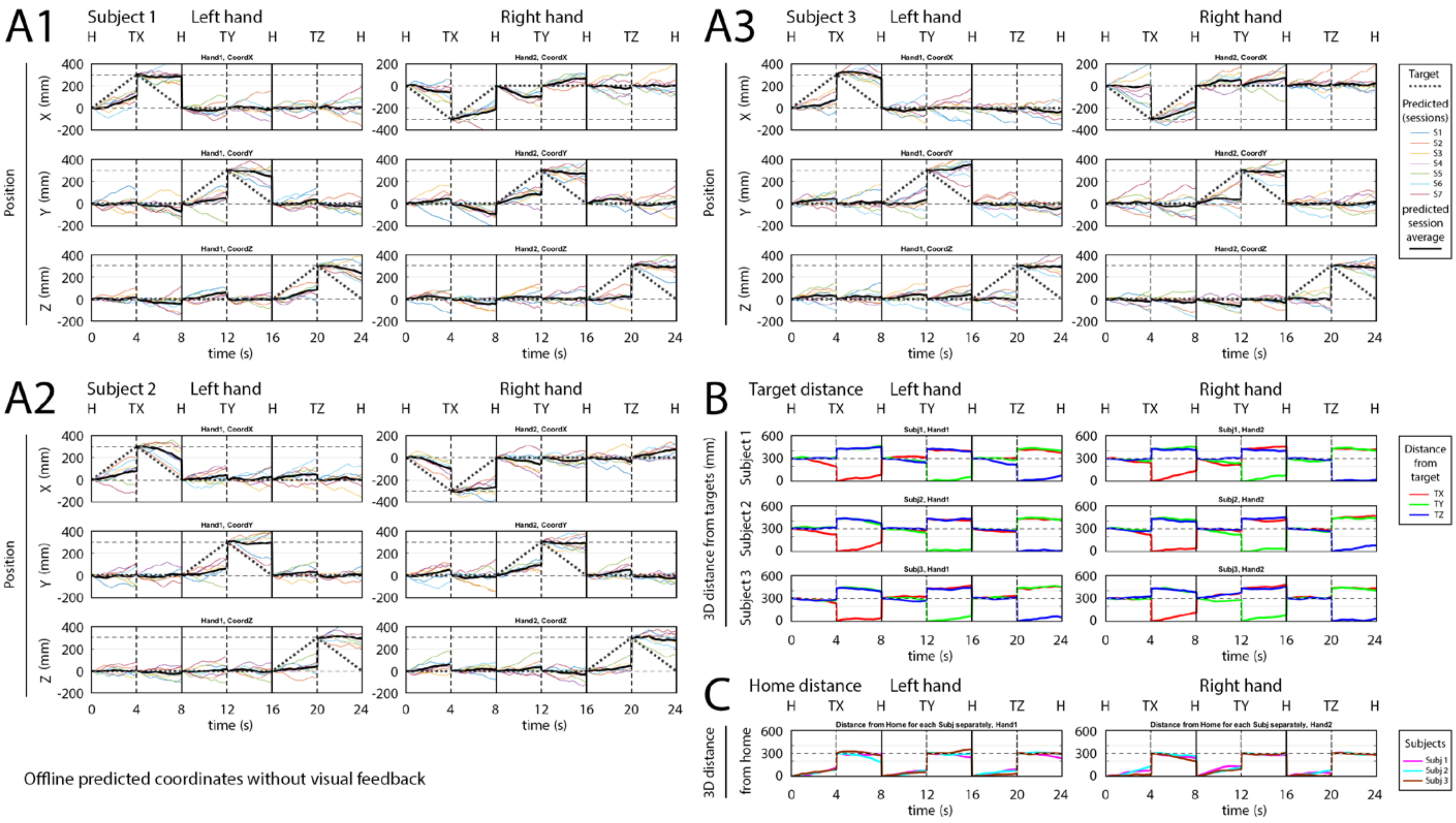

Supplementary Figure 1. Evaluation chart for predicted coordinates resulting from offline MTP experiments without visual feedback. Label H-TX-H-TY-H-TZ-H for each sub-plot indicates the corresponding task over time (H: home, T: target). **(A):** a comparison of predicted and target trajectories in three spatial dimensions. **(B-C):** time-varying distance between the 3D location of the predicted hand position and each of the three targets **(B)**, and home positions **(C)**.

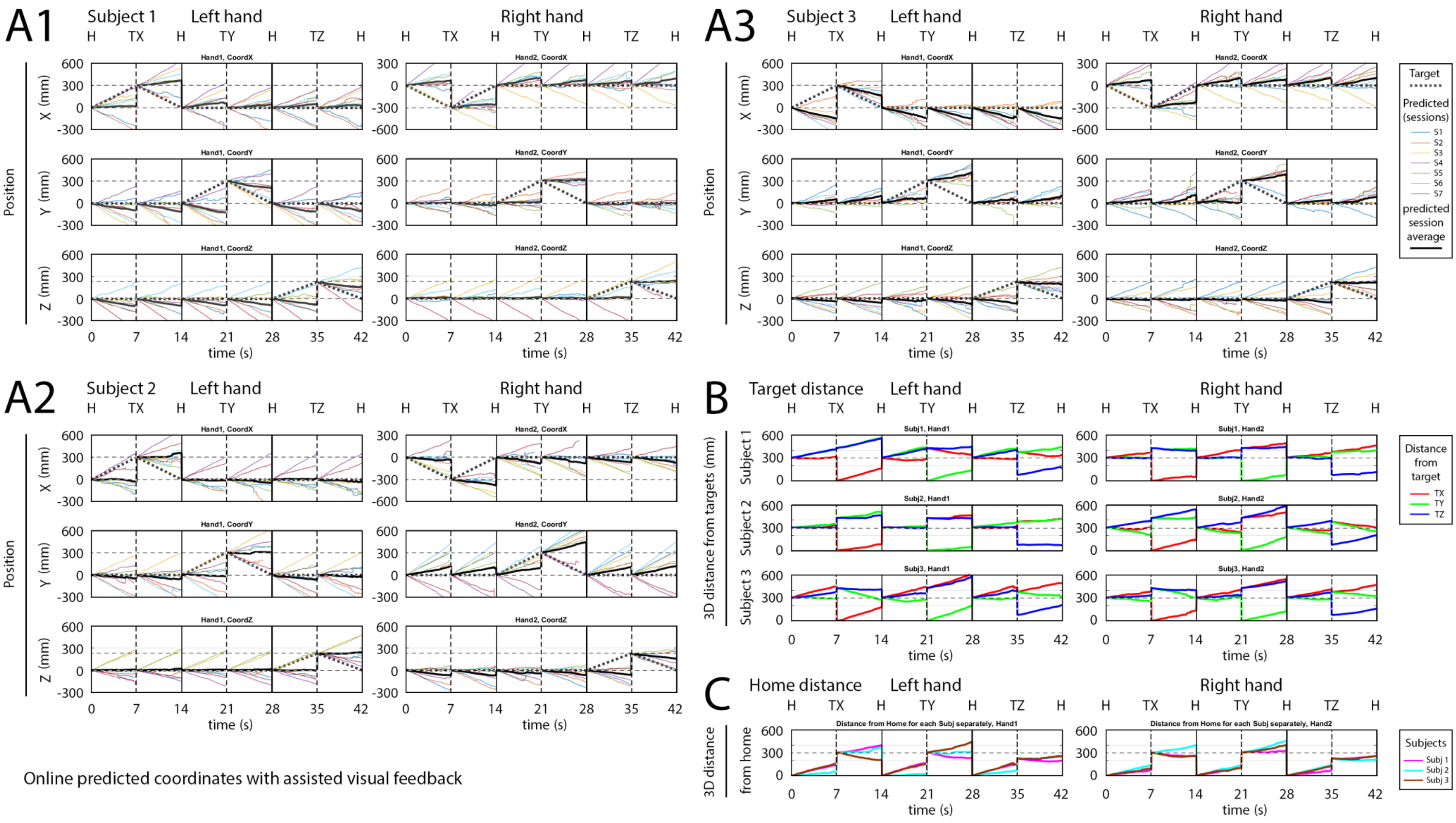

Supplementary Figure 2. Evaluation chart for predicted coordinates resulting from online MTP experiments using assisted visual feedback. Label H-TX-H-TY-H-TZ-H for each sub-plot indicates the corresponding task over time (H: home, T: target). **(A):** a comparison of predicted and target trajectories in three spatial dimensions. **(B-C):** time-varying distance between the 3D location of the predicted hand position and each of the three targets **(B)**, and home positions **(C)**.

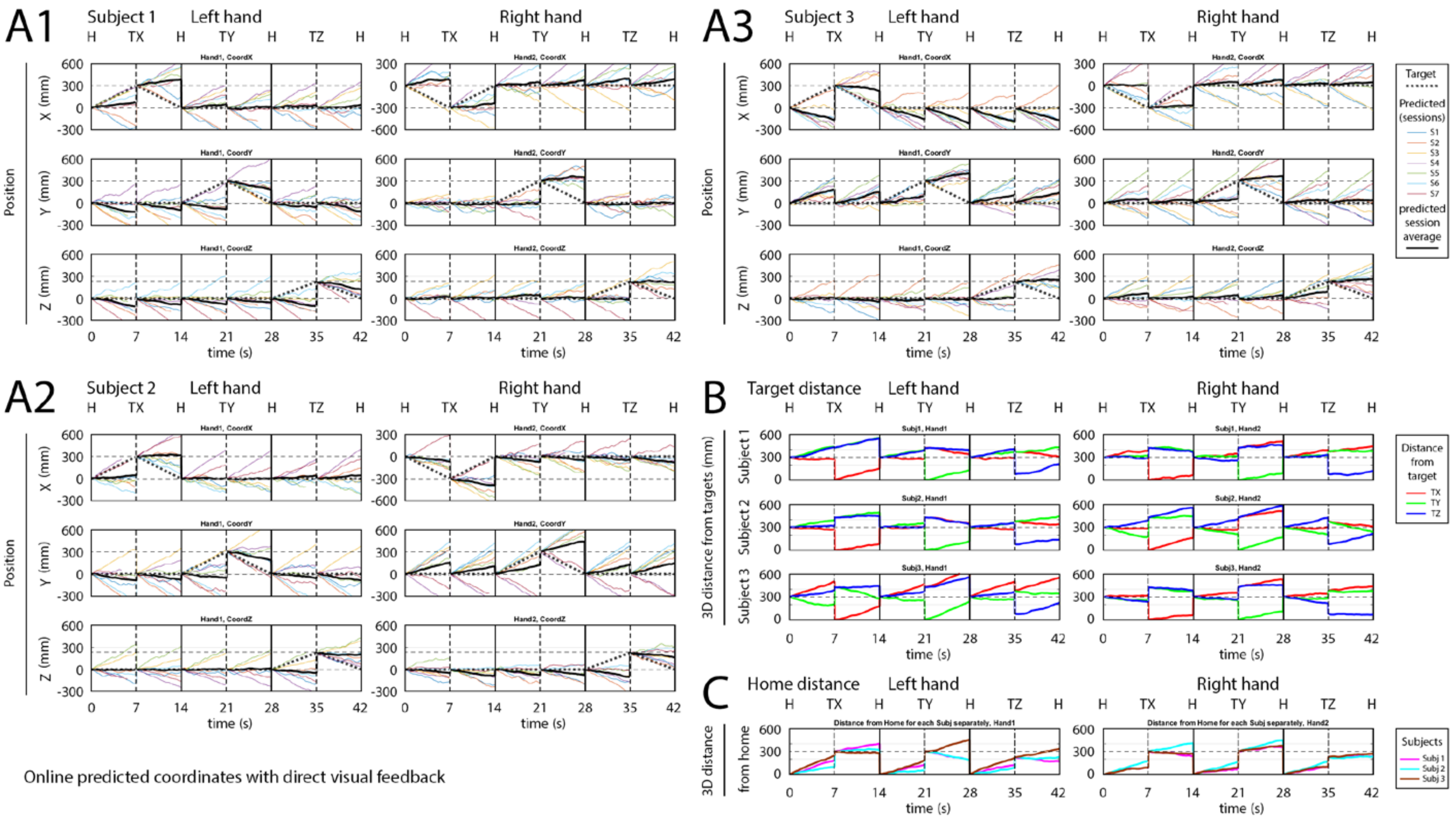

Supplementary Figure 3. Evaluation chart for predicted coordinates resulting from online MTP using direct visual feedback. Label H-TX-H-TY-H-TZ-H for each sub-plot indicates the corresponding task over time (H: home, T: target). **(A):** a comparison of predicted and target trajectories in three spatial dimensions. **(B-C):** time-varying distance between the 3D location of the predicted hand position and each of the three targets **(B)**, and home positions **(C)**.

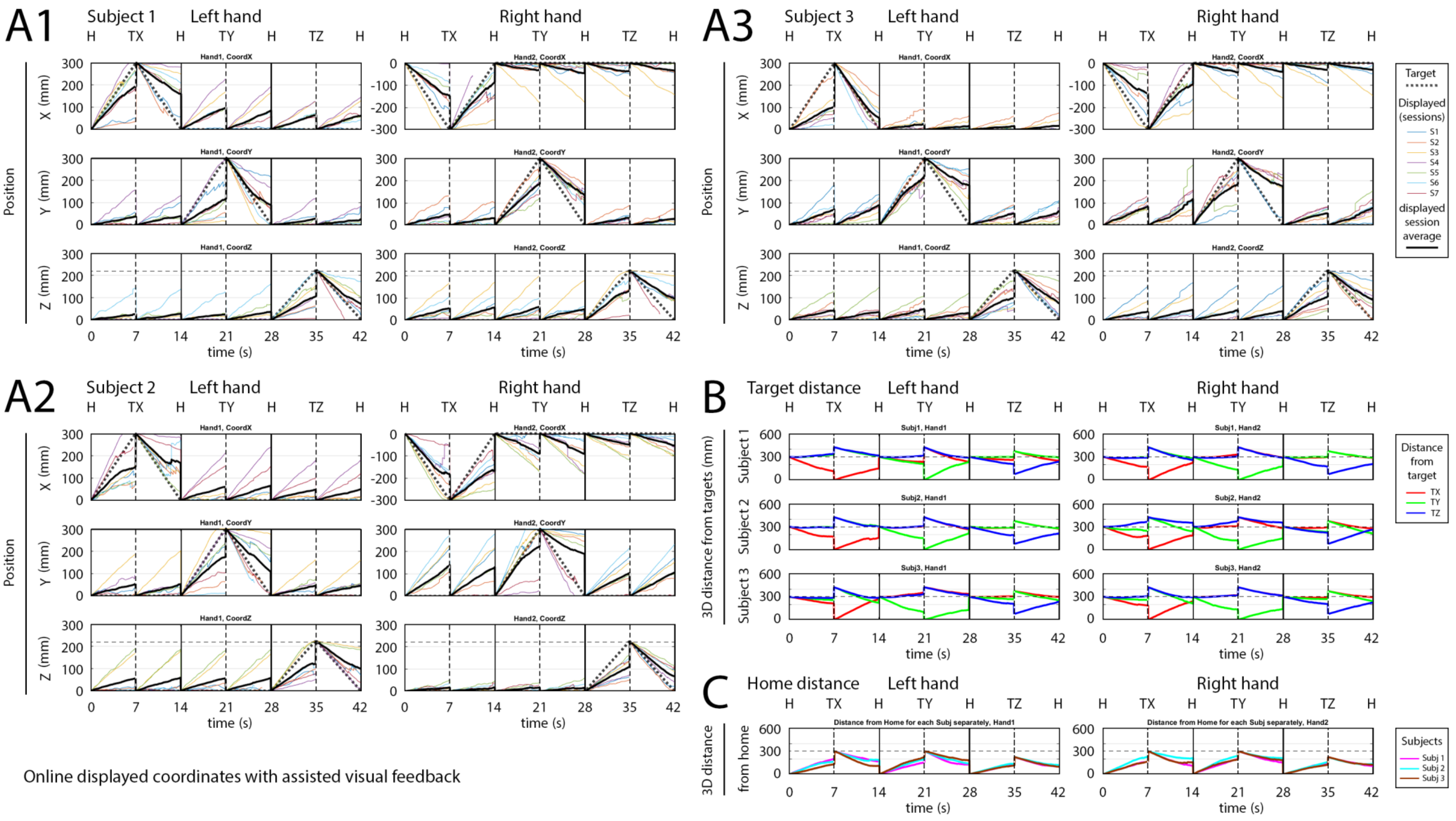

Supplementary Figure 4. Evaluation chart for displayed coordinates resulting from online MTP experiments using assisted visual feedback. Label H-TX-H-TY-H-TZ-H for each sub-plot indicates the corresponding task over time (H: home, T: target). **(A):** a comparison of displayed and target trajectories in three spatial dimensions. **(B-C):** time-varying distance between the 3D location of the displayed hand position and each of the three targets **(B)**, and home positions **(C)**.

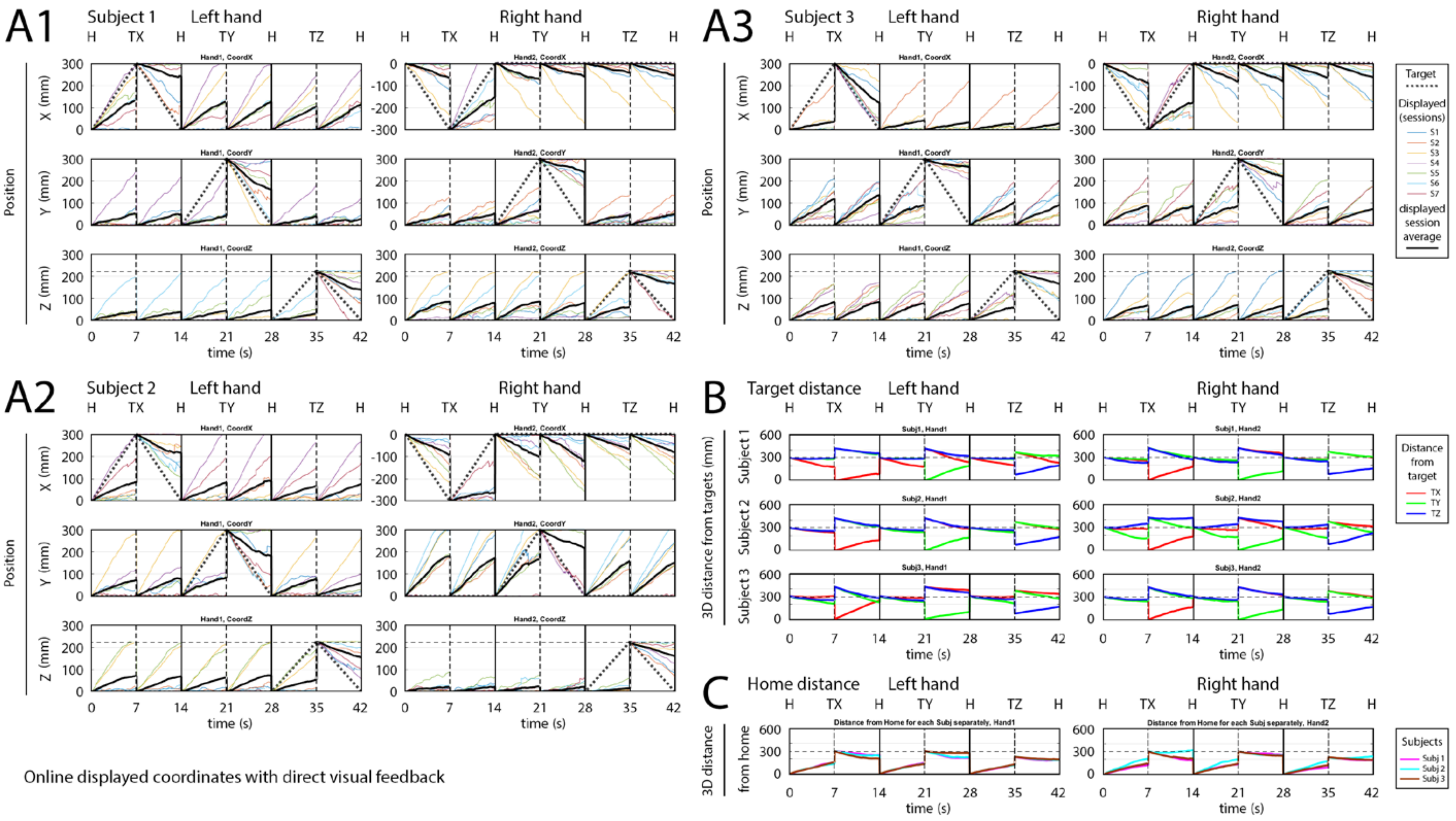

Supplementary Figure 5. Evaluation chart for displayed coordinates resulting from online MTP experiments using direct visual feedback. Label H-TX-H-TY-H-TZ-H for each sub-plot indicates the corresponding task over time (H: home, T: target). **(A):** a comparison of displayed and target trajectories in three spatial dimensions. **(B-C):** time-varying distance between the 3D location of the displayed hand position and each of the three targets **(B)**, and home positions **(C)**.
